# Speech Perception and Localisation with SCORE Bimodal: A Loudness Normalisation Strategy for Combined Cochlear Implant and Hearing Aid Stimulation

**DOI:** 10.1371/journal.pone.0045385

**Published:** 2012-10-24

**Authors:** Tom Francart, Hugh McDermott

**Affiliations:** 1 ExpORL, Department of Neurosciences, Katholieke Universiteit Leuven, Leuven, Belgium; 2 The Bionics Institute, Melbourne, Victoria, Australia; 3 The University of Melbourne, Melbourne, Victoria, Australia; UNLV, United States of America

## Abstract

A significant fraction of newly implanted cochlear implant recipients use a hearing aid in their non-implanted ear. SCORE bimodal is a sound processing strategy developed for this configuration, aimed at normalising loudness perception and improving binaural loudness balance. Speech perception performance in quiet and noise and sound localisation ability of six bimodal listeners were measured with and without application of SCORE. Speech perception in quiet was measured either with only acoustic, only electric, or bimodal stimulation, at soft and normal conversational levels. For speech in quiet there was a significant improvement with application of SCORE. Speech perception in noise was measured for either steady-state noise, fluctuating noise, or a competing talker, at conversational levels with bimodal stimulation. For speech in noise there was no significant effect of application of SCORE. Modelling of interaural loudness differences in a long-term-average-speech-spectrum-weighted click train indicated that left-right discrimination of sound sources can improve with application of SCORE. As SCORE was found to leave speech perception unaffected or to improve it, it seems suitable for implementation in clinical devices.

## Introduction

Many newly implanted cochlear implant (CI) recipients have residual hearing in the non-implanted ear. Bimodal stimulation, i.e., the combination of a CI in one ear and acoustic stimulation in the other, has several advantages compared to a unilateral CI [1], [2], such as improved speech perception in noise. Unfortunately, there are no CIs and hearing aids (HAs) specifically designed for combined use. This leads to suboptimal perception of binaural cues [3] and binaural balance [4], poor sound-source localisation ability and probably reduced wearing comfort. Additionally, there are no generally accepted and scientifically validated fitting methods for bimodal devices, and fitting is often performed at different locations, e.g., the CI clinic and the HA dispenser.

Recently, we developed the SCORE (Stimulus Control to Optimise Recipient Experience) bimodal signal processing strategy [4], [5], which normalises loudness using models of loudness perception that run in real-time on the two devices. Normal-hearing loudness is estimated at microphone level for each ear, hearing-impaired and CI loudness are estimated after hearing aid and CI speech processing respectively, and the overall output level is adjusted to equalise the loudness of the final outputs to the normal hearing ones. SCORE bimodal has been shown to improve binaural balance [4]. Unilateral SCORE (CI-only) had been shown to improve speech perception in quiet at low levels [5]. While SCORE bimodal normalises loudness and improves binaural balance, these factors are subordinate to speech perception. Therefore, in the current study we assessed the effect of SCORE on speech perception in quiet and in noise.

While bimodal listeners are stimulated binaurally and could therefore in principle have access to binaural cues (interaural time and level differences), they generally exhibit poor sound-source localisation ability. This is due to reduced sensitivity to binaural cues [3], [6], and also to the processing in current commercial devices which often deteriorates binaural cues. Here, we will focus on interaural level difference (ILD) cues. While in the literature on binaural sound localisation the term ILD is often used to indicate both the physical measure and the perceptual effect, we think that for listeners with asymmetric hearing losses the concept of interaural loudness difference (ILoD) makes more sense. The binaural system does not have access to level differences per se, but to the corresponding neural activation, which is closely related to loudness. For ILoDs by themselves to be useful for sound source localisation, there needs to be a consistent relation between location and ILoD, i.e., they need to be consistent across signal bandwidths and stimulation levels. For typical bimodal listeners this is often not the case: loudness growth for electric and acoustic hearing is quite different [7] and the high-frequency part of broadband sounds can often not be perceived acoustically. Depending on the individual listener's hearing loss and CI fitting, ILoDs may be different for the same sound at different levels, or for sounds at the same level but with different bandwidths. As SCORE aims to normalise loudness perception at the two ears, we expect that it may be beneficial for sound-source localisation using ILoDs. We investigated the effect of SCORE on sound-source localisation with simulations using loudness models and preliminary localisation tests with two listeners.

## Methods

Three experiments were performed: speech perception in quiet, speech perception in noise, and sound-source localisation, all using either clinical processing (termed ACE) and the processing under investigation (SCORE). In what follows we first describe the two processing strategies and then provide details of the procedures used.

### ACE and SCORE

**Figure 1:**
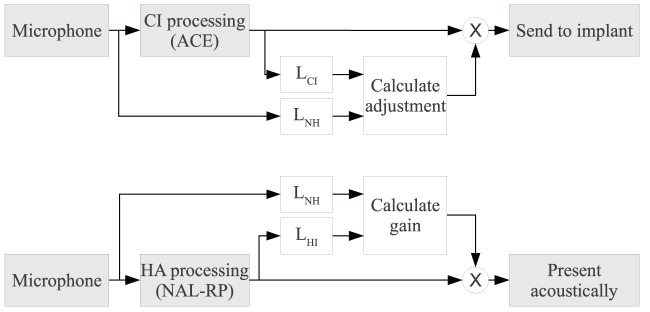
Block diagram of ACE and SCORE processing. The grey blocks indicate the clinical ACE processing and the white blocks the add-on SCORE processing.*L_NH_*, *L_HI_*, and *L_CI_* indicate respectively loudness models for normal hearing, impaired hearing and cochlear implant (electrical) stimulation.

Two sound-processing strategies were contrasted: ACE and SCORE. By ACE we mean the clinically used processing for the electrically stimulated ear and a linear HA fitted according to the NAL-RP rule [8] for the acoustically stimulated ear. The signal processing for ACE and SCORE is described in detail by [4]. Here we will briefly summarise the important points. A block diagram of the two strategies is shown in figure 1. The grey blocks indicate the clinical ACE processing and the white blocks the add-on SCORE processing.

The ACE strategy [9], [10] is implemented in Cochlear's commercial sound processors, which were used by the subjects on a daily basis. For the current study all sound processing was implemented in Matlab, using the Nucleus Matlab Toolbox provided by Cochlear, plus a simulation of the microphone characteristics of the Freedom processor. The HA was simulated by filtering the acoustic signal in Matlab according to the desired frequency response.

SCORE is add-on processing to any sound processing strategy, and in this case was added onto ACE. It uses models of loudness perception [11], [12] to estimate on the one hand the loudness experienced by a normal-hearing listener when listening to the signals received by the microphones at each ear (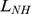 in the figure), and on the other hand the loudness experienced by a hearing-impaired listener when listening to the signal at the output of the hearing aid (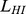), and of a CI listener when listening to the signal at the output of the sound processor (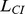). The overall levels of the acoustic and electric stimulus were adjusted by SCORE such that the estimated loudness at the output of both devices was the same as the normal-hearing loudness at the input.

For the estimate of normal-hearing loudness at the microphone and impaired-hearing loudness at the HA output, the loudness model described by [12] was used. Its parameters were set based on the unaided audiogram of each listener.

For electric stimulation a simplified version of the model described by [11] was used. For each electrical pulse a loudness contribution was calculated, and the total loudness was estimated as the sum of pulse loudness contributions over a certain period of time. In the published model the pulse loudness contribution is given by 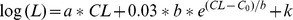, with 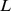 the loudness contribution in linear units, 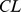 the current level (in clinical current units) of the pulse, and parameters 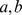, and 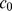, which potentially have different values for each electrode. In the simplified model proposed by [5], this relationship is simplified to 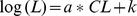, where 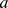 (the slope of the loudness growth function) is determined in a monaural loudness balancing experiment between stimuli of different bandwidths, and 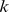 is a scaling factor. The formula for the level adjustment of the electric signal is 
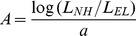
, with 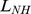 the loudness estimate of the normal-hearing loudness model, 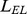 the loudness estimate of the electric loudness model, and 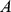 the adjustment in fraction of the dynamic range. Dynamic range is defined as follows: During fitting of the CI sound processor, for each electrode a just-audible threshold (T) and comfortable loudness (C) level are determined with a pulse train of usually 900 pps. In the final stages of ACE processing, filtered acoustic signals with magnitudes between 0 and 1 are mapped between the T and C levels for each electrode. The dynamic range is the difference between C and T level for each electrode. In SCORE, electric adjustments are expressed as a fraction of the dynamic range; e.g., an adjustment of 0.1 would mean that for each electrode (C-T)*0.1 current units are added to each pulse. 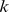 only has effect on 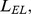 so the overall effect of 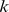 is to add a fixed value to each adjustment calculated. For the speech perception experiments reported later, 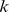 was determined in a preliminary loudness balancing experiment (see below).

Adjustments in dB (for the acoustic signal) and in fraction of the dynamic range (for the electric signal) were calculated for each frame of 6.9 ms duration. Adjustments were smoothed using a set of heuristics and automatic-gain-control-like processing with attack and release times of 5 ms and 50 ms respectively. Note that no automatic gain control or other compression was used.

As SCORE operated on the broadband acoustic signal and affected all electric channels equally, it did not affect the spectral characteristics of the signals. While the operation of SCORE depended very much on individual characteristics of hearing loss and the input signals used, generally its application had the following effects: at the electric side SCORE counteracted loudness artefacts introduced by maxima selection, and at the acoustic side SCORE counteracted the effect of reduced audibility of high-frequency sounds due to a lack of high frequency residual hearing. This often had the effect that soft phonemes were amplified.

### Apparatus

Electric and acoustic stimulation was done under direct computer control, using the APEX 3 program developed at ExpORL, K.U.Leuven [13]. The subjects' own HAs and sound processors were not used. For acoustic stimulation, an RME Fireface 400 sound card (Audio AG, Haimhausen, Germany) was used, and for electric stimulation the Cochlear NICv2 interface connected to an L34 experimental processor. The clocks of the sound card and L34 were synchronised. The electric stimulus was delayed by 1.5 ms relative to the acoustic stimulus to compensate for the estimated average acoustic travelling-wave delay [3]. For acoustic stimulation an Etymotic ER-3A insert earphone was driven by the sound card. The insert phone was calibrated using a HA-1 2cc coupler. All signal processing and stimulus preparation was done using Matlab (The Mathworks, Natick, MA, USA).

### Subjects and fitting

Six subjects were recruited who also participated in our previous SCORE study [4]. They used a CI and contralateral HA on a daily basis. Their audiograms and other relevant information are shown in table 1. Permission to conduct the studies was obtained from the Human Research and Ethics Committee of the Royal Victorian Eye and Ear Hospital, and each participant provided written informed consent.

**Table 1:** Subject details.

Subject	Age (y)	CI use (y)	CI side	Aetiology	Unaided threshold (dB HL)					
					250	500	1000	2000	4000	8000Hz
S26	75	1.7	R	Unknown	60	65	70	75	85	75
S27	78	2.4	L	Unknown	75	70	65	60	60	100
S30	60	2.6	R	Unknown	30	55	100	–	–	–
S31	62	1.7	R	Unknown	70	75	90	80	105	–
S32	54	7.2	R	Menieres	70	85	80	80	80	100
S34	53	2.6	R	Unknown	50	60	85	–	–	–

“Age” is in years at the time of testing. “CI use” is the number of years of implant use at the time of testing. “CI side” is left (L) or right (R); the HA was on the other side. Unaided pure-tone thresholds are given in dB HL. Unmeasurable thresholds are indicated by a dash.

For the CI sound processor, the clinical fitting was used, with the volume set to a level that provided comfortable listening for running speech presented at 60 dB A. SCORE requires parameter 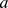 to be determined during fitting. We used the value previously determined using loudness balancing experiments for these six subjects [4]. For the HA, target aided thresholds were calculated according to the NAL-RP rule [8]. Aided thresholds were measured through the simulated HA and gains were adjusted to attain the target aided thresholds. The overall gain was adjusted to obtain a balanced percept for speech at 60 dB A.

### Procedures

**Figure 2:**
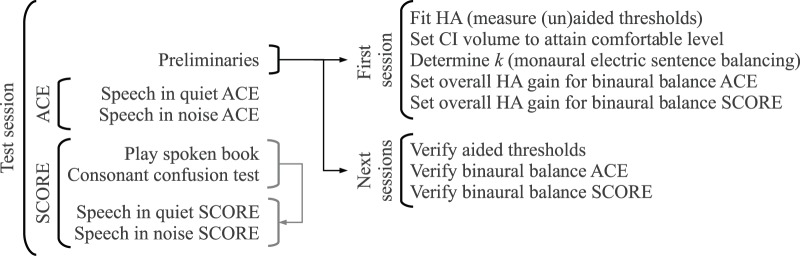
Schematic overview of a test session.

A schematic overview of a test session is shown in figure 2. At the beginning of a session, some preliminary tests were done. Then a block of either ACE or SCORE tests was completed. SCORE tests were always preceded by some training, consisting of listening to an audiobook and performing a consonant confusion test with feedback. ACE tests were not preceded by training, as each of the subjects used ACE on a daily basis. In pilot tests, no learning effect was found in the ACE condition. When asked, the subjects could not hear a difference between their own processor and the experimental implementation of ACE. In what follows we describe each of these steps in detail.

#### Preliminaries

At the beginning of the first test session, the HA was fitted according to the NAL-RP rule. The volume of the CI was set to attain a comfortable and well-audible percept, comparable to the loudness obtained with the subject's own speech processor for sounds at a normal conversational level. The electric loudness model used by SCORE has a parameter 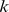 that influences the average stimulation level adjustment that will be applied. In order to conduct a fair comparison between ACE and SCORE, loudness balancing was done with only electric stimulation using a sentence processed by ACE or SCORE, adjusting the 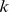 parameter for equal loudness. The sentence was “I like that song”, uttered by a female speaker at a level of 60 dB A. In each trial the sentence was presented twice, once processed by ACE and once by SCORE, in random order, and the subject was asked which interval sounded louder overall, the first or the second. 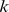 was adapted in a 1-up/1-down adaptive procedure, in steps that corresponded to a level difference of 10% of the dynamic range. The procedure was stopped after 10 reversals and the resulting value of 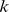 was calculated as the mean of its values at the last 6 reversals. This procedure was run twice and the final value for 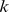 was calculated as the mean of those two runs.

**Figure 3:**
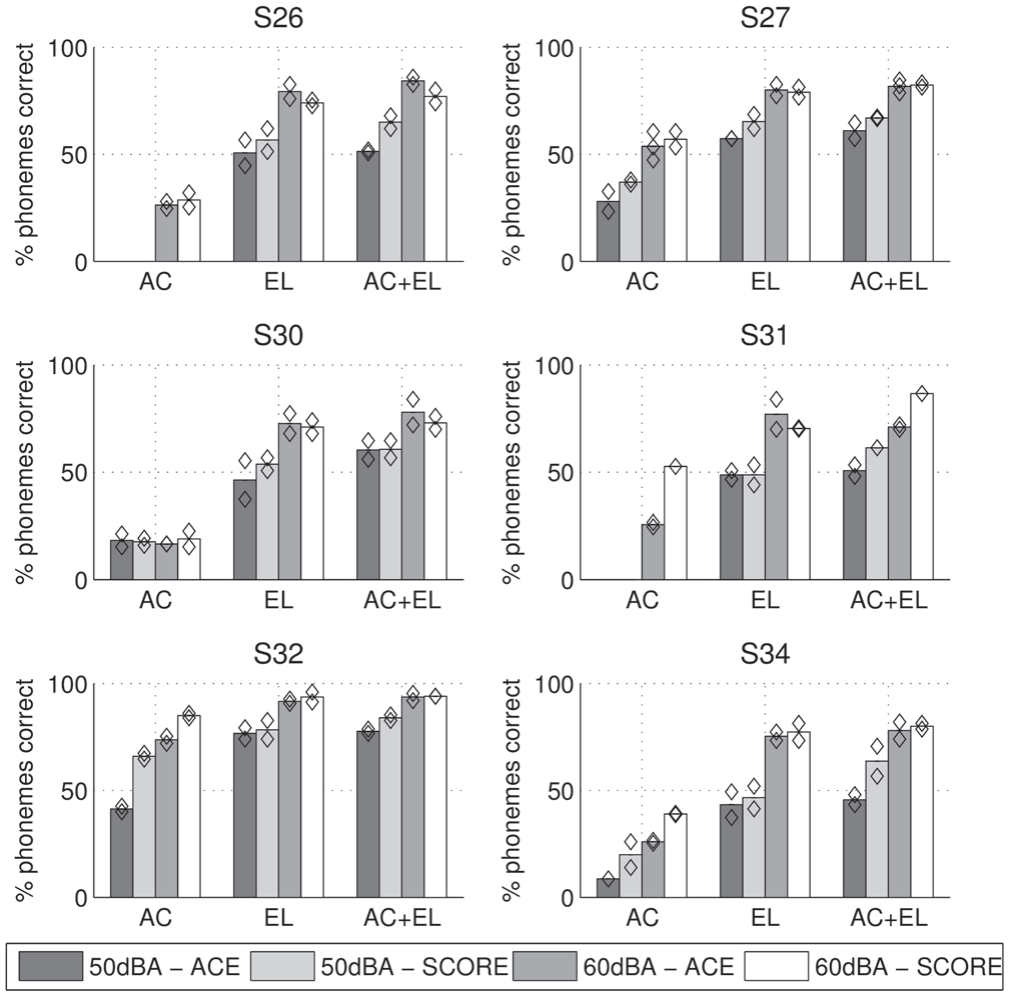
Speech in quiet results for ACE and SCORE, at 50 and 60 dB A presentation level, either acoustic-only (AC), electric-only (EL), or bimodal (AC+EL) for each subject. Diamonds indicate results for individual lists.

Then binaural loudness balancing was performed between electric and acoustic stimulation, separately for ACE and SCORE. For SCORE the 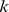 value determined in the balancing experiment was used. The experimenter adjusted the overall gain of the HA to obtain a balanced percept. The subjects were asked if they perceived a single fused sound image, or rather a separate sound image for each ear. If there was a single sound image, they were asked to indicate for which overall gain the stimulus sounded in the centre of the head. If there were two sound images, they were asked to indicate for which overall gain the stimulus sounded equally loud in the two ears. Three out of six subjects perceived a clearly fused sound image. The stimuli at the input of the ACE or SCORE processing were 1-s long long-term average speech spectrum weighted noise, according to ANSI-S3.5 [14], and recorded sentences uttered by a male and female speaker at an average level of 60 dB A. Usually the same overall HA gain was found for all three stimuli. If this was not the case, the average gain across these three stimuli was used.

At the beginning of all test sessions after the first one, aided thresholds were verified and always found to be within 5 dB of the target. Binaural balance was also verified, and the overall gain was always found to be within 4 dB of the previous measurement. Therefore the same fitting and overall gain for the HA was used in all test sessions.

While the subjects used the ACE strategy on a daily basis, they were initially unfamiliar with the SCORE strategy. In order not to give ACE an unfair advantage due to familiarity, ideally SCORE would have to be implemented on a wearable platform, and the subjects would be given extensive take-home listening experience. Because this was not feasible at this stage of the development of SCORE due to technical and time constraints, we only performed acute experiments but conducted some brief training with SCORE immediately before each block of SCORE speech tests. Note that such brief training is probably insufficient, so when interpreting our results, one should take into account that performance with SCORE was probably worse than it would be after extensive familiarisation. The training consisted of 1) listening to an audiobook for around 3 minutes, while following the transcription, and 2) a closed-set consonant identification test. In the consonant identification test 16 consonants were used, embedded in the vowel/a:/, uttered by a male speaker. All 16 possibilities were shown on a computer screen, and the subject was asked to indicate the one they heard. After each trial feedback was given and the correct response was indicated. Each consonant was presented three times and the whole test was run twice. Note that the results are not presented in the current paper because this experiment only served as training.

#### Speech in quiet

Speech understanding in quiet was measured using lists of 50 consonant-nucleus-consonant words, uttered by an Australian female speaker. A full factorial 2×3×2 design was used with factors presentation level (50 or 60 dB A), modality (acoustic (AC), electric (EL), or bimodal (AC+EL)), and processing scheme (ACE or SCORE). Levels of each factor were presented in random order, except for processing scheme. The order of the ACE and SCORE conditions was counterbalanced across subjects and test sessions. For each condition two lists were used in different test sessions. Before each list, ten consonant-nucleus-consonant trials from another list were conducted to familiarise the subject with the condition. The results of those ten trials were discarded. There were 40 lists available in total and list-subject-condition assignments were counterbalanced. For each word the phoneme score out of three was determined and finally a percent-phonemes-correct score was calculated for the entire list. Subjects were instructed to repeat whatever they heard, even if only part of a word, or a non-lexical word.

#### Speech in noise

Speech understanding in noise was measured using the BKB-like [15] lists of 16 sentences, uttered by an Australian female speaker. Sentences were presented bimodally at 60 dB A with one of three maskers. A full factorial 3×2 design was used with factors masker (LTASS, ICRA, or CT; see below), and processing scheme (ACE or SCORE). Levels of the masker factor were randomised. The order of the ACE and SCORE conditions was counterbalanced across subjects and test sessions. For each condition two lists were used. The masker was either a steady noise shaped according to the long-time-average speech spectrum (LTASS), IRCA-5 fluctuating noise (ICRA), or a male competing talker (CT) in Swedish. The last two maskers are described in detail by [16]. Briefly, the ICRA-5 noise contained silent gaps of maximally 250 ms duration, and the Swedish competing-talker signal was uttered by a male speaker and temporal gaps were limited to 100 ms. The speech reception threshold (SRT) was determined by keeping the level of the speech constant at 60 dB A and adapting the level of the noise in a 1-up/1-down adaptive procedure in steps of 2 dB. A sentence was considered correct only if all keywords were repeated correctly in the correct order. The SRT was calculated as the mean of the last 8 signal-to-noise ratios. The standard deviation of this mean was always smaller than 4 dB.

#### Localisation

Preliminary tests of sound-source localisation were conducted with two subjects. The input signal was a click train with a fundamental frequency of 400 Hz, filtered according to the international long-term average speech spectrum [14]. This signal was filtered using head-related transfer functions measured through the Freedom CI sound processor on an artificial head. The subjects performed a closed-set sound direction identification task in the horizontal plane, with angles ranging from −45 degrees to +45 degrees, in steps of 15 degrees, with 0 degrees corresponding to straight in front of the listener. In a first condition, the input signal was set at a fixed level of 60 dB A and was presented three times from each direction. In a second condition, level roving was used: the input signal was set randomly at 50, 55, or 60 dB A, and was presented three times for each level and from each direction. Before starting the localisation experiment, the stimulus from 0 degrees was binaurally balanced, individually for ACE and SCORE.

Additionally we used loudness models to assess the interaural loudness differences available with these signals for each subject and for angles of incidence ranging from -90 degrees to +90 degrees. We used the loudness models described in section “ACE and SCORE”, with their parameters set according to the subjects' individual pure tone audiograms and CI fittings.

## Results

### Speech in quiet

**Figure 4:**
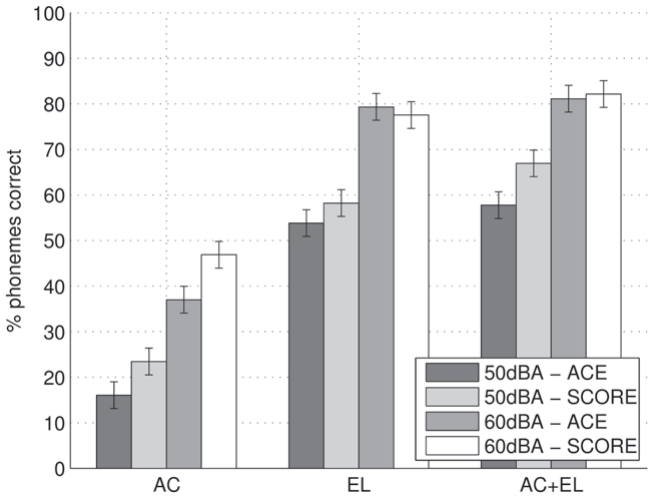
Speech in quiet results averaged across subjects. The total error bar length indicates Fletcher's least significant difference.

Individual phoneme scores for speech in quiet are shown in figure 3 and average scores across subjects are shown in figure 4. Test-retest differences were small, with an average of 5 percentage points in the phoneme score. We performed a repeated-measures ANOVA of percent phonemes correct with factors modality (AC, EL, or AC+EL), level (50 or 60 dB A), and processing scheme (ACE or SCORE). A Greenhouse-Geisser correction was applied to correct for non-sphericity of factor modality. There was a significant effect of modality (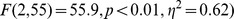, a significant improvement with increasing level (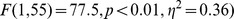, a significant improvement of SCORE over ACE (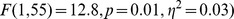, and no significant interactions, including the interaction between processing scheme and modality (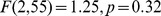). In the conditions with electric stimulation (EL and AC+EL) the effect was largest at 50 dB SPL. On average, across all subjects and conditions performance was 5 percentage points better for SCORE than for ACE.

When considering individual results, SCORE never clearly decreased performance (i.e., there was no performance deterioration larger than the test-retest reliability), except for S26 in the EL and EL+AC conditions at 60 dB A. While this could be an effect of insufficient training, it should also be noted that in our previous loudness-balancing study [4], the results for S26 were much worse than average, presumably because his fitting was quite different than that of the other subjects, owing to facial nerve stimulation at higher stimulation levels. Application of SCORE may therefore not be desirable for this subject.

### Speech in noise

**Figure 5:**
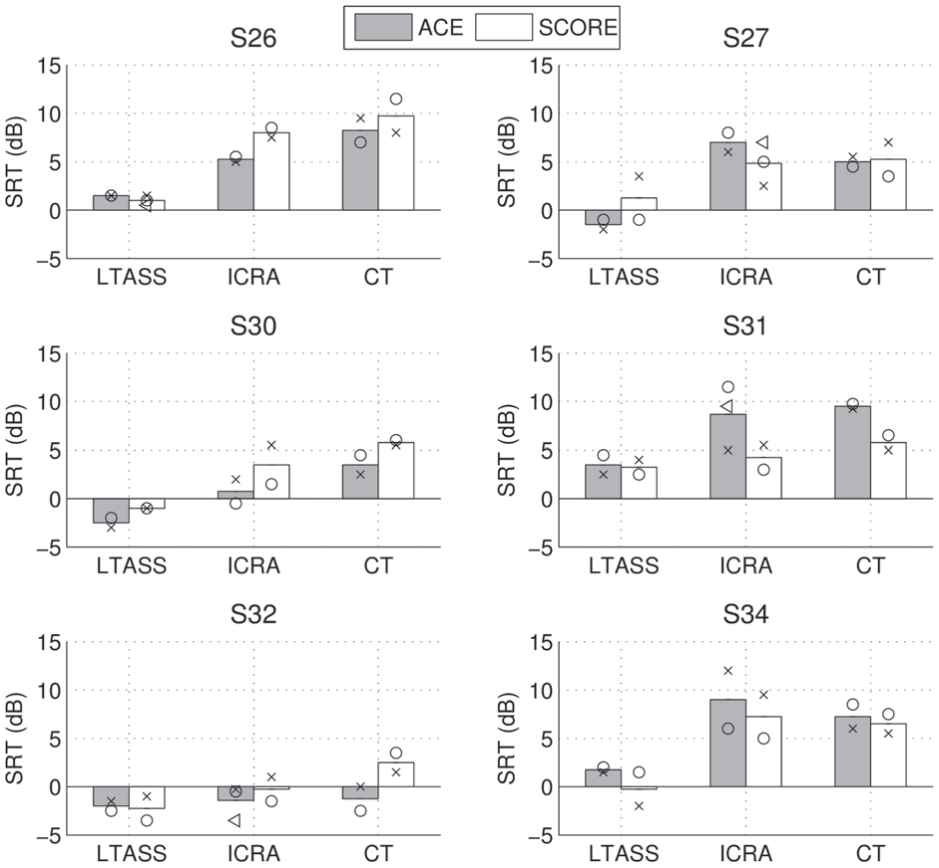
Speech in noise results for ACE and SCORE, for a steady state noise masker (LTASS), fluctuating noise (ICRA), and a competing talker (CT), for each subject. The markers indicate SRTs calculated from a single list, and markers with the same symbols indicate results obtained during the same test session.

**Figure 6:**
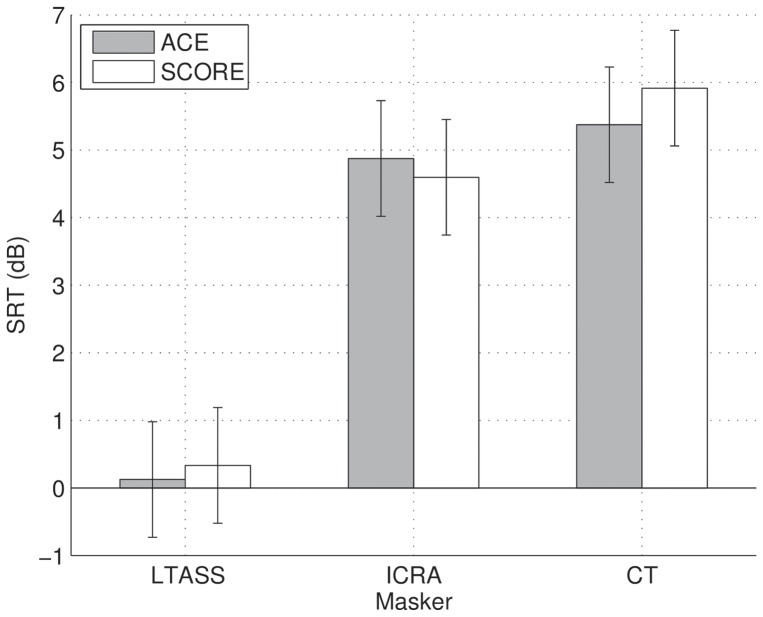
Speech in noise results averaged across subjects. The total error bar length indicates Fletcher's least significant difference.

Individual phoneme scores for speech in noise are shown in figure 5 and average scores across subjects are shown in figure 6. We performed a repeated-measures ANOVA of SRT with factors masker (LTASS, ICRA, or CT), and processing scheme (ACE or SCORE). A Greenhouse-Geisser correction was applied to correct for non-sphericity of factor masker. There was a significant effect of masker (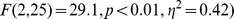, no effect of processing scheme (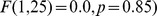, and no effect of the interaction between masker and processing scheme (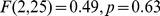). The difference in SRT between ACE and SCORE lies with 90% confidence between −1.1 (worse performance with SCORE) and 0.8 dB (better performance with SCORE).

### Sound source localisation

In this section we will first present interaural level and loudness differences in the stimulus, calculated using loudness models (see section “ACE and SCORE” above). Then we will show the results of the sound source localisation experiments and compare with the modelling results.

#### Modelling

**Figure 7:**
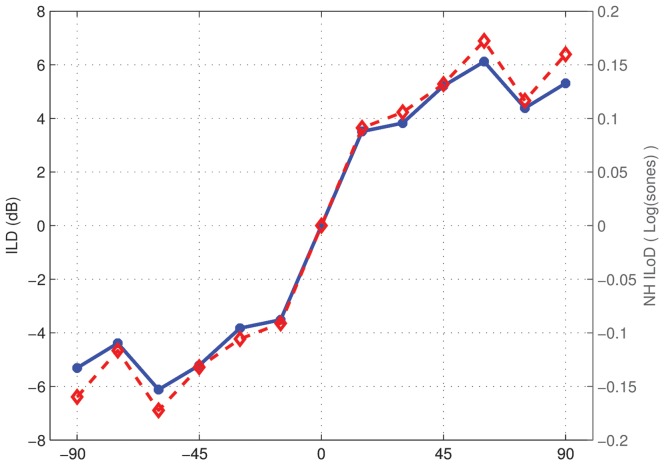
Interaural level and loudness differences for the speech-weighted click train used in the localisation experiments and simulations. ILDs in dB are shown in blue with round markers and the corresponding interaural loudness differences for a normal-hearing listener are shown in red with diamond markers.

In what follows a long-term-average-speech-spectrum weighted click train was used as the input stimulus. In the figure 7 the ILD of the stimulus at the microphone is shown. The corresponding ILoDs for a normal-hearing listener are also shown, as calculated using the normal-hearing loudness model for a stimulus at 60 dB A. Normal-hearing ILoDs at lower levels were very similar. ILDs and corresponding ILoDs are small: the maximal ILD value is 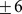 dB. This means that if no other cues are available for localisation, we can expect poor performance, as just-noticeable differences in ILD for normal-hearing listeners are in the order of 1 dB [17] and for bimodal listeners in the order of 1.7 dB [6]. The ILD function is also non-monotonic beyond 45 degrees. This means that it is not possible to distinguish between angles larger than 45 degrees on one side using only ILD cues. Normal-hearing listeners are able to do this using additional cues (interaural time differences and monaural spectral cues). As normal-hearing listeners have the same loudness growth function for both ears, and the loudness growth function is approximately linear for the current range of levels used, the ILoD follows the ILD closely. This illustrates why for normal-hearing listeners the term ILD and ILoD can be used interchangeably.

**Figure 8:**
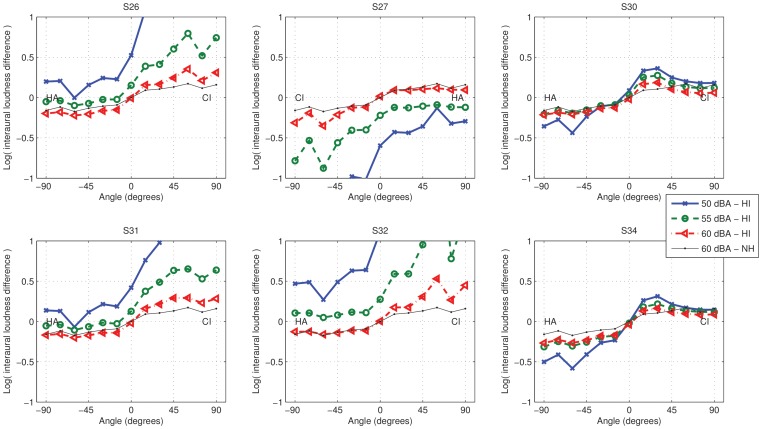
Interaural loudness differences for a speech-weighted click train, predicted according to the hearing loss of each participant. The thick coloured lines indicate ILoDs at the output of ACE for different input levels. The thin black line shows the ILoD for a normal-hearing listener for a stimulus at 60 dB A. The thick lines show the loudness after CI and HA processing, taking into account hearing impairment.

In figure 8, ILoDs are shown for the hearing-impaired bimodal listeners who took part in the current study, calculated with the loudness models configured using their audiograms and CI fitting parameters. The overall level of the acoustic signal was adjusted to obtain loudness balance for the stimulus from 0 degrees at 60 dB A. ILoDs were calculated for the input signal at three different levels: 50, 55, and 60 dB A. ILoDs for a normal-hearing listener are shown in each panel as a reference. SCORE has the effect of changing ILoDs to the normal-hearing ones. The resulting patterns for our hearing-impaired listeners can be broken down into two categories: first, for S26, S27, S31, and S32, who had residual hearing at frequencies beyond 3 kHz, ILoDs for the 60 dB A stimulus follow the normal-hearing one quite closely. While there are some differences, these would probably not have a large perceptual effect. At lower levels, though, the ILoD functions are shifted to the CI side, which means that at these levels the stimulus will be lateralised to the HA side, irrespective of angle of incidence. We expect that the application of SCORE in this case might not lead to good sound-source localisation, as ILoDs are small, but would lead to correct left-right discrimination. The remaining two subjects, S30 and S34, only had measurable residual hearing below 3 kHz. In this case ILoDs increased with decreasing level, but an extra non-monotonicity was introduced in the ILoD function at around 15–30 degrees. While increased ILoDs could improve localisation, this is hampered by the fact that ILoDs are inconsistent across levels: the auditory system presumably “expects” the same ILoD for the same spectrum at different levels. In this case we do not expect the application of SCORE to improve left-right discrimination, but the reduction of the non-monotonicity at 15–30 degrees could improve localisation at those angles.

In summary, depending of the signal's spectral content and the subject's audiogram, different ILoD-versus-angle patterns are possible. Assuming that bimodal listeners only have access to ILoD cues, and not to interaural time differences or detailed monaural spectral cues, localisation of the filtered click train stimulus with a spectrum similar to speech, will be poor. We expect that application of SCORE will lead to improved left-right discrimination, which is an important ability in real life, for some subjects.

#### Experimental results

**Figure 9:**
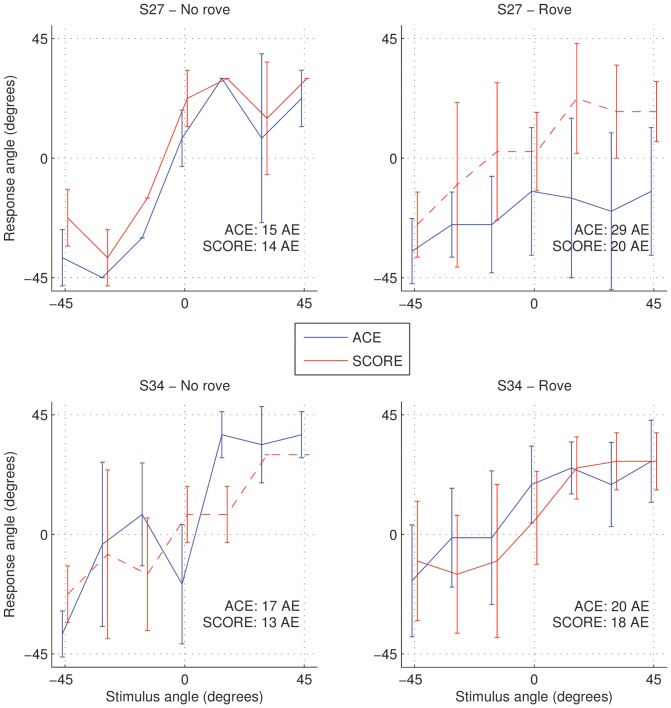
Localisation results with ACE and SCORE with and without level roving. For each angle the average response and corresponding standard deviations are shown. The overall absolute localisation error (AE) in degrees is shown for each condition.

We performed a localisation experiment with one subject from each group: S27 and S32. S27 had residual hearing beyond 3 kHz and S32 did not. We only presented stimuli from angles smaller than 45 degrees, because of the non-monotonicity in the ILD-versus-angle function. In figure 9 the localisation results for S27 and S34 are shown. For S27 without roving, there was essentially no difference between ACE and SCORE. This corresponds to the observation that the hearing-impaired ILoD in figure 8 follows the normal-hearing one. For S27 with roving, there was a big difference. Further analysis of the data (not shown) indicated that for levels lower than 60 dB A, the subject always lateralised the stimulus to the HA side, which again corresponds to the observation that the ILoDs in figure 8 for 50 and 55 dB A are below zero for all angles of incidence.

For S34 without roving, the main difference between ACE and SCORE occurred at 15 degrees. In figure 8 there is a corresponding non-monotonicity (peak) at 15 degrees in the hearing-impaired functions, which was removed by SCORE. For S34 with roving, there was no clear difference between ACE and SCORE. This can be explained by two counteracting effects. In the ACE condition, larger ILoDs were available at low levels, which improved left-right lateralisation, yielding an advantage of ACE over SCORE. On the other hand, in the SCORE condition ILoDs were presumably more consistent across levels, yielding an advantage for SCORE. Note that overall performance for both subjects was very poor, even though the stimulus from 0 degrees was balanced at 60 dB A, which would not be the case for many stimuli with standard clinical processing. Generally these subjects were able to discriminate between left and right, but could not make a finer distinction between angles of incidence.

## Discussion

The SCORE strategy was designed to normalise loudness and improve binaural balance with bimodal stimulation [4]. In the current study we measured the effect of SCORE on speech perception and sound-source localisation ability. The expected outcome was no change in speech perception and potentially small improvements in localisation. For speech perception in quiet we found an improvement with application of SCORE, and for speech perception in noise we found no change between ACE and SCORE. For localisation we expect improved left-right discrimination with application of SCORE, based on modelling results.

Our experiments were all acute: the subjects had no experience with SCORE outside of the laboratory, and only very limited experience within the laboratory. To achieve maximal performance with a new signal-processing scheme, usually extensive familiarisation is required. Therefore our results should be considered a worst-case scenario in this respect. While some initial training was given by playing an audiobook and conducting a consonant identification test, this only provided the most basic familiarisation. In pilot tests with S27 we noticed clear differences before and after this training stage, which indicates that some familiarisation is required.

The performance improvement with SCORE in quiet is consistent with a previous study of speech perception in quiet at soft levels, with unilateral electric stimulation only, in which a significant improvement was found with application of SCORE [5]. The improvement is probably due to increased audibility of soft phonemes. In noise this effect is reduced because SCORE operates on overall loudness and therefore does not increase the level of soft phonemes plus noise as much. Note that the use of different speech materials (words versus sentences) for tests in quiet and noise is a confounding factor when assessing the effect of SCORE: theoretically this could mean that SCORE was only beneficial for individual words and not for sentences. However, given that its physical effect is the same irrespective of the material, this seems unlikely. When considering individual results for speech perception in noise, there were no differences between ACE and SCORE for the steady-state masker (LTASS), but sometimes large differences for the fluctuating maskers. Presumably this was at least partly caused by increased variability for fluctuating maskers [16], but could also have been due in part to familiarisation issues. However, on average, across subjects there was no significant effect of processing scheme. The same inter subject variability is often seen in studies assessing the effect of hearing aid compression parameters [18], [19]. For speech in noise, average performance was much worse for the fluctuating maskers (CT and ICRA), which is consistent with the literature for CI listeners [20]. This indicates that our subjects were not able to use the temporal gaps in the masker to understand speech, like normal-hearing listeners do [16], but rather were more distracted by the fluctuations.

From the comparison between our sound-source localisation experiments and assessment of ILoDs calculated using loudness models, it follows that SCORE provided improvements when expected. However, it should be noted that overall, using either ACE or SCORE, localisation performance for these subjects was very poor. This is due to their inability to use interaural time difference cues and the limited extent of ILDs available in the signal (up to 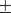
5 dB, see figure 8 for angles between −45 and 45 degrees). Given that just-noticeable differences in ILD of bimodal listeners are in the order of 1.7 dB [6], it is not surprising that their localisation performance is poor, even if ILoDs comparable to normal-hearing ones are available. SCORE is clearly not a complete solution for bimodal sound-source localisation. A combination of SCORE with a recently developed ILD enhancement algorithm [21] could provide a solution.

While the acoustic processing in SCORE might seem similar to typical HA compression systems, there are several important differences. First of all, the SCORE processing aims to normalise loudness. While loudness normalisation is also part of common HA fitting methods such as the NAL and DSL prescriptions [22], it is only a secondary goal, and only works for average signals and average subjects. Note that if loudness normalisation is not desired, SCORE can easily be configured to transform the target (normal-hearing) loudness to the desired function. A second difference between SCORE and standard compression is that SCORE normalises the total loudness, instead of operating independently in several frequency bands. This means that even if part of a broadband sound cannot be perceived acoustically, the overall loudness of the sound in the impaired ear will be adjusted to the loudness of the broadband sound for a normal-hearing listener, retaining the normal ILoD. A third difference is that SCORE utilises models of loudness in real-time, yielding an estimate of loudness that is much more precise than can be achieved by setting the parameters of a compression system during fitting.

In conclusion, SCORE processing did not deteriorate speech perception in quiet or in noise, for stationary and fluctuating maskers, and improved speech perception in quiet. SCORE normalises interaural loudness differences, which can be beneficial for sound-source localisation with bimodal hearing.
